# Quality and barriers of outpatient diabetes care in rural health facilities in Uganda – a mixed methods study

**DOI:** 10.1186/s12913-019-4535-x

**Published:** 2019-10-16

**Authors:** Catherine Birabwa, Mulekya F. Bwambale, Peter Waiswa, Roy W. Mayega

**Affiliations:** 1 Department of Health Policy, Planning and Management, Makerere University Kampala – College of Health Sciences School of Public Health, P.O. Box 7072, Kampala, Uganda; 2 Department of Epidemiology and Biostatistics, Makerere University Kampala – College of Health Sciences School of Public Health, P.O. Box 7072, Kampala, Uganda

**Keywords:** Type 2 diabetes, Quality of care, Barriers, Health facilities, Rural Uganda

## Abstract

**Background:**

Despite the increasing burden of diabetes in Uganda, little is known about the quality of type 2 diabetes mellitus (T2DM) care especially in rural areas. Poor quality of care is a serious limitation to the control of diabetes and its complications. This study assessed the quality of care and barriers to service delivery in two rural districts in Eastern Uganda.

**Methods:**

This was a mixed methods cross-sectional study, conducted in six facilities. A randomly selected sample of 377 people with diabetes was interviewed using a pre-tested interviewer administered questionnaire. Key informant interviews were also conducted with diabetes care providers. Data was collected on health outcomes, processes of care and foundations for high quality health systems. The study included three health outcomes, six elements of competent care under processes and 16 elements of tools/resources and workforce under foundations. Descriptive statistics were computed to determine performance under each domain, and thematic content analysis was used for qualitative data.

**Results:**

The mean age of participants was 49 years (±11.7 years) with a median duration of diabetes of 4 years (inter-quartile range = 2.7 years). The overall facility readiness score was 73.9%. Inadequacies were found in health worker training in standard diabetes care, availability of medicines, and management systems for services. These were also the key barriers to provision and access to care in addition to lack of affordability. Screening of clients for blood cholesterol and microvascular complications was very low. Regarding outcomes; 56.8% of participants had controlled blood glucose, 49.3% had controlled blood pressure; and 84.0% reported having at least one complication.

**Conclusion:**

The quality of T2DM care provided in these rural facilities is sub-optimal, especially the process of care. The consequences include sub-optimal blood glucose and blood pressure control. Improving availability of essential medicines and basic technologies and competence of health workers can improve the care process leading to better outcomes.

## Background

Type 2 Diabetes Mellitus (T2DM) is among the leading causes of mortality and ill-health worldwide, accounting for over one million deaths [1, 2]. Sub- Saharan Africa (SSA) is no exception, as reports show an increase in the prevalence of diabetes over the years [3]. Uganda’s reported overall prevalence of T2DM is relatively low at 1.4% [4]. However, studies have revealed several issues of concern including high levels of unawareness of hyperglycaemic status among people with hyperglycaemia [4], existence of pockets of high prevalence in some geographical regions [5, 6] and under-diagnosis [5]. Global estimates show that the greatest increase in the burden of T2DM will occur in low- and middle- income countries (LMICs) [7] and if not addressed, the adverse physical and socio-economic consequences of diabetes will constrain the health systems in these countries [3].

The rising prevalence of diseases like diabetes creates a pressing need for high quality health systems to optimise healthcare particularly in resource-limited settings. Health systems in LMICs are only beginning to adapt to chronic care and therefore ill-prepared to handle the rising burden of diabetes and its complications [3, 8]. This is due to health systems gaps including scarcity of diagnostic and monitoring equipment, inadequately knowledgeable and skilled healthcare providers, lack of appropriate guidelines, insufficient facilities to manage complication, limited access to medicines and poor integration of diabetes services [3, 9]. These bottlenecks result in, misdiagnosis, untimeliness of care, poor patient retention and coordination; which not only contribute to mortality and morbidity, but also result in wastage of resources, catastrophic expenditures and health-related suffering [10, 11].

High quality health systems provide care that optimizes health outcomes, are valued and trusted by the people and respond to evolving population needs [10]. Good quality care conforms to national or international standards [12]; and based on the report by Kruk and colleagues, quality health systems comprise of three main domains: quality impacts (health outcomes, confidence in the system and economic benefits); processes of care (competent care and user experience); and foundations for the system, including the population served, governance, platforms for service delivery, the work force, tools and resources [10]. This study assessed the elements of health outcomes, evidence-based care and health prevention including screening for complications, health workforce, tools and resources.

In Uganda, interventions towards improving diabetes or chronic care were initiated such as capacity building within the public sector; collaborations between different sectors, development partners and civil society organizations and promotion of integrated management of chronic diseases [13, 14]. However, care for chronic diseases is persistently affected by poor availability of equipment and essential medicines, limited diagnostic capacity, inadequate essential services and lack of standard guidelines [15, 16]. Similar gaps affect readiness of health systems in other developing countries like Ethiopia [17], Bangladesh [18] and Malawi [19].

Countable studies have assessed processes of care or quality impacts for diabetes care in Uganda. One study conducted in an urban hospital found low rates of blood glucose (fasting plasma glucose< 7.2 mmol/l) and blood pressure control at 42.8 and 56% respectively; as well as low screening rates for microvascular complications [20]. Processes of care and health outcomes have also been found to be poor in other developing countries. In Brazil, adequate glycemic control (glycosylated haemoglobin HbA1c < 7%) was found in only 18.7% of the participants [19].

There’s limited available knowledge and information on the quality of T2DM care in health facilities from predominantly rural settings, where 76% of Uganda’s population lives [21]. As Uganda’s health system increasingly adapts to the growing problem of non-communicable diseases (NCDs), there’s need to understand the gaps in diabetes care in rural health facilities so that they can be appropriately addressed to ensure that good quality care is available & accessible to all affected people.

This study assessed the quality of diabetes care in rural health facilities in Uganda and also explored barriers to the provision of and access to quality diabetes healthcare. This is imperative for diabetes care planning and delivery in rural settings where the urgency for health systems to address NCDs is high but evidence to guide decisions is limited.

## Methods

### Study design and setting

A concurrent embedded mixed-methods study was conducted, in which qualitative data was nested in the largely quantitative cross-sectional study. This enabled us to have a broader understanding of diabetes care in rural settings, by corroborating results of facility audit and client survey, with those from provider interviews.

This facility-based study was conducted in two district hospitals and four Health Centre IVs (HCIVs) in Iganga and Mayuge districts, Eastern Uganda. Uganda implements a level-based healthcare system for public health services [22] that includes the Health Centre II (at parish level) that serves about 5000 people; Health Centre III (at sub-county level) that is an intermediate facility serving about 25,000 people. The HC IVs and district hospitals are referral units serving about 100,000 people at Health Sub-district and district levels. They have medical officers and provide referral services. Both Iganga and Mayuge districts have one general hospital and two HC IVs each, in addition to other lower-level facilities. Of the six health facilities included in this study, only one hospital had a dedicated diabetes clinic. In the other health facilities, diabetes clients were seen on a general out-patients basis on any day of the week. Previous studies in the study districts found high prevalence of T2DM (7.4%) and its risk factors among people aged 35 years and older [6, 23].

### Study population and selection

The target population for this study were people with T2DM. Using Kish’s formula (Kish, 1965), a sample of 377 T2DM clients were sampled and allocated to the six study facilities proportionate to the average number of registered diabetes patients in each district within two months prior to the study, based on the district health information system 2 and facility records (Table 1).

**Table 1:** Sample size determination for diabetes clients

*By district*			
District	No. of diabetes clients seen (*N*)		Required sample size X = (N/T)*n, where *n* = 377
District A	264		289
District B	80		87.7 ≈ 88
Total (T)	344		377
*By facility*			
Health facility	District	No. of diabetes clients seen (n’)	Required sample size =(n’/N)*X
HF -A	A	18	19.7
HF -B	A	36	39.4
HF -C	B	25	27.5
HF -D	B	25	27.5
HF -E	A	210	229.9
HF -F	B	30	33

*HF=Health facility.*

The health facilities in which the study was conducted were purposively selected based on the level of provision of key diabetes services. This study focused on the HC IVs and hospitals because more comprehensive diabetes services are provided at these levels. From these facilities, participants were selected using systematic sampling from the diabetes registers in the respective health facilities. Only adult clients aged 18 years and above who had known their diabetes status for at least one year and provided written consent were enlisted in the study. We excluded clients that were too ill to provide required information.

### Data collection and measurements

The assessment was conducted between March and June 2016 by a team of 8 trained research assistants using pre-tested tools. The research assistants conducted face-to-face interviews, direct observation and records review. The data collection tools used for the assessment were developed by the authors based on key constructs from the Service Availability and Readiness Assessment (SARA) manual and the International Diabetes Federation (IDF) T2DM guidelines. The sub-components of the three dimensions were transformed into questions or checklist items (Additional file 1).

The participant questionnaire was developed to obtain information on background characteristics (e.g. sex, age, education level, source of livelihood, illness history and comorbidities), process of care (e.g. diabetes education and counselling, monitoring of blood glucose and pressure, and screening for microvascular complications), and health outcomes (blood glucose level, blood pressure and presence of any chronic complication). The questionnaire also included open ended questions to document challenges experienced by participants in accessing diabetes services.

A service-specific readiness assessment was conducted for the 6 health facilities using a checklist to collect data on the foundations of quality systems for diabetes care. These included equipment and supplies (e.g. adult weighing scale, blood pressure machine, blood glucose machine, glucose and urine test strips, insulin syringes, measuring tape, Snellen charts and guidelines); essential medicines (e.g. metformin, glibenclamide and insulin), and diagnostic capacity (e.g. blood glucose, cholesterol, urine protein and albuminuria).

The quality of diabetes care indicators were categorized into the three domains of the framework by Kruk and colleagues (Table 2). The elements under foundations of the system comprised of 16 tracer items categorized into four domains: a) staff & training (two items), b) basic technologies (seven items), c) diagnostics (four items) and d) essential medicines (three items). These were selected based on the SARA guidelines [24]. In addition, two indicators were included to assess support functions for diabetes care delivery. These were support supervision and information system, assessed as whether or not the facility had received support supervision within 6 months prior to study and if they had a specific data-capture system for T2DM services respectively.

**Table 2:** Study indicators and measurement

Dimension	Indicators	Definition
Foundations of quality systems	*Staff and training*	
	Guidelines for diagnosis & treatment of DM	Observed presence of national (&other) guidelines for DM
	Staff trained in diabetes diagnosis & treatment	At least one staff member providing DM services trained in some aspect of DM care
	*Basic technologies/Equipment*	
	Adult weighing scale	Observed availability & reported functionality of each item at the facility
	Blood pressure measurement device	
	Stethoscope	
	Glucometer	
	Blood glucose test strips	
	Measuring tape	
	Urine protein test strips	
	*Essential medicines*	
	Metformin	Observed availability of each medicine at the facility
	Glibenclamide	
	Insulin	
	*Diagnostics*	
	Blood glucose	Able to conduct the test at the facility and observed availability of functioning equipment & reagents for the test
	Urine dipstick- protein	
	Urine dipstick- albumin/ketones	
	Blood cholesterol	
Processes of care	Blood glucose monitoring	Received at least one glucose measurement in past year
	Blood pressure monitoring	Received at least one blood pressure measurement in past year
	Blood cholesterol monitoring	Received at least one cholesterol measurement in past year
	Monitoring kidney disease	Received urine-protein test in past year
	Eye examination	Received dilated eye examination in past year
	Foot examination	Received foot examination in past year
Health Outcomes	Blood glucose control	Having RBS level of ≤11.0 mmol/l
	Blood pressure control	Having BP of ≤130/80 mmHg
	Chronic complications	Having at least one of the selected complications

The processes of care consisted of a) presence of blood glucose, b) blood pressure and c) blood cholesterol monitoring, d) monitoring of kidney disease, and e) routine eye and foot examinations, based on the International Diabetes Federation guidelines for T2DM [25] (Table 2). These were assessed using client interviews through recall and review of client medical records. The respondent was asked if they had received a given test within 12 months prior to the study (yes/no).

The health outcomes included a) controlled blood glucose, b) blood pressure and c) presence of at least one chronic complication of T2DM. Blood glucose control (defined as Random Blood Sugar (RBS) ≤ 11.1 mmol/L) was assessed using the blood glucose measurement taken on the day the respondent was interviewed or the most recent measurement (a value within three months). Blood pressure control (defined as BP ≤ 130/80 mmHg) was assessed using the measurement taken on the day the respondent was interviewed. This was taken after a client had rested for at least 15 min and recorded in the patients’ medical records. Cut offs were based on guidelines [25, 26]. Chronic complications were measured using self-reports & records review. Complications explored were foot lesions (ulcers/amputations), eye lesions (visual impairment/blindness) and peripheral neuropathy (Table 2). Data on these clinical outcomes for each individual were transcribed from their medical records into the relevant section of the questionnaire.

The questions in the tools were reviewed by three co-investigators to affirm their face and content validity. The questionnaire and checklist were then pre-tested in two health facilities in Kampala district, with the purpose of ensuring that the health workers and diabetes clients had better understanding of the study questions. The clients’ questionnaire was translated into the local language (Lusoga), by an experienced research assistant who was fluent in the local language. The questionnaire was then translated back to English, with emphasis on conceptual equivalence. Inconsistencies were resolved through consultations with another research assistant.

### Data management and analysis

Quantitative data was entered in pre-designed data entry screens using EpiData (v3.1), cleaned and exported to STATA (v12) for analysis. Univariate analysis was done to summarize data from the structured observations using counts; to describe performance under each quality of care dimension and to summarize survey information using mean, standard deviation, median and interquartile range for continuous variables like age, blood glucose levels and duration of T2DM; as well as frequencies & proportions for categorical variables such as education level and presence of comorbidities or complications.

Performance under each dimension was computed separately. The structural-measures were summarized as the service readiness score (mean availability of the tracer items) under the four specified domains using SARA guidelines. The total number of items available (from the facility assessment) under each domain was computed. This was then divided by the required number of items in each category and multiplied by 100. The service readiness score was then computed as the mean score of the four domains. The process and outcome of care scores were computed as a proportion for each of the selected tests and outcome respectively.

### Qualitative data

We conducted key informant interviews with health workers from the six study facilities where clients were recruited. These were purposively selected based on their level of involvement in the provision of diabetes services at the facilities. The objective of these interviews was to obtain the health providers’ perspective on diabetes care in rural settings.

A key informant interview guide was developed by the researchers that was used to obtain information from the health workers. The guide included questions exploring the health worker’s opinion on quality of diabetes care, process of care and challenges faced in providing diabetes services in rural centres. The interviews with health workers were conducted concurrently with the client survey data collection.

Data from key informant interviews was analysed using content analysis as described by Graneheim and Lundman [27]. The audio-recorded interviews were transcribed (in English) into word documents. The transcripts were first reviewed to familiarize with the data and make preliminary observations. This was followed by a more detailed review of the transcripts to identify patterns and meanings that were used to develop a coding scheme. The codes were then applied to the data in each transcript. Responses with similar codes were re-categorized under a unifying theme. Each theme was then described and used to generate the narrative for the question of interest. The data is presented in text with anonymized quotes.

Responses to the open-ended question in the client survey were tallied to determine the most important challenges affecting access to diabetes services in the health facilities.

## Results

### Participants’ background characteristics

Of the 377 participants, 62.1% were female. The mean age of participants was 49 (±11.7) years. Close to 20.0% had no formal education. Over three quarters were married/living together and most (51.2%) were peasant farmers. The median duration of diabetes among participants was 4 years (IQR = 2.7). Close to three-quarters of participants (272; 72.2%) had at least one diabetes related comorbidity. Details of participants’ background characteristics are summarized in Table 3.

**Table 3:** Background characteristics of study participants

Characteristic	Category	Total (N = 377)
Age	< 30	14 (3.71)
	30–39	49 (13.00)
	40–49	131 (34.75)
	50–59	108 (28.65)
	≥60	75 (19.89)
Education	No formal education	60 (15.9)
	Primary	200 (53.1)
	Secondary	92 (24.4)
	Institution	25 (6.6)
Religion	Catholic	54 (14.32)
	Protestant	157 (41.65)
	Muslim	142 (37.67)
	Pentecostal	22 (5.84)
	Others	2 (0.53)
Marital status	Never married	14 (3.7)
	Married/living together	297 (78.8)
	Widowed/separated	66 (17.5)
Source of livelihood	Not working	74 (19.63)
	Salary/wage earner	61 (16.18)
	Peasant farmer	193 (51.19)
	Self-employed	49 (13.00)
Duration of diabetes	1 to 5 yrs	248 (65.8)
	Above 5 yrs	129 (34.2)
Having any comorbidity	No	105 (27.85)
	Yes	272 (72.15)

For the qualitative phase, we interviewed health workers that were most involved in the provision of diabetes services. These were eight [8] health workers, who included 2 medical doctors, 4 clinical officers and two nurses (Table 4).

**Table 4:** Characteristics of health workers

Respondent ID	Age group	Education level	Cadre	Duration of work at facility
01	20–29	Diploma clinical medicine	Clinical officer	3 months
02	50–59	University	Nursing officer	8 years
03	30–39	Diploma clinical medicine	Clinical officer	4 years
04	20–29	University	Medical doctor	2 years
05	20–29	Certificate	Nurse	3 years
06	20–29	Diploma clinical medicine	Clinical officer	2.5 years
07	20–29	Diploma clinical medicine	Clinical officer	1.5 years
08	40–49	University	Medical doctor	10 years

### Quality of type 2 diabetes care

#### Assessment of foundations of a quality system

The overall capacity of health facilities to provide diabetes care was 73.9%. This was higher in the hospitals (84.7%) compared to the HC IVs (68.5%). Diagnostic capacity at all six health facilities was adequate except for blood cholesterol that was available at one facility, a hospital. Only one facility had at least one health worker involved in diabetes care that had been trained in standard T2DM care. Only two of the six health facilities had all the three tracer medicines for diabetes care. Half of the facilities (3/6) did not have Metformin, which is the standard first line treatment for T2DM. All facilities had not received any support supervision related to diabetes from the Ministry of Health or district health officials in the six months prior to the study; half of the facilities (3/6) had a T2DM specific documentation system. The findings on structural performance per domain are summarized in Table 5.

**Table 5:** Quality of diabetes care

Dimension	measures	Overall	HC IVs	Hospitals
Foundations	Staff & training	58.3%	50%	75%
	Basic technologies	85.7%	82.1%	92.9%
	Diagnostics	79.2%	75%	87.5%
	Essential medicines	72.2%	66.7%	83.4%
Process measures	Blood glucose	100%(377/377)	100%(377/377)	100%(377/377)
	BP	100%(377/377)	100%(377/377)	100%(377/377)
	Cholesterol	0%(0/377)	0%(0/377)	0%(0/377)
	Urine Protein	6.4%(24/377)	22.2%(24/108)	0%(0/269)
	Eye exam	9.0%(34/377)	23.1%(25/108)	3.4%(9/269)
	Foot exam	8.8%(33/377)	20.4%(22/108)	4.1%(11/269)
Outcome measures	Glucose control	56.8%(214/377)	57.4%(62/108)	56.5%(152/269)
	BP control	49.3%(186/377)	62%(67/108)	44.2%(119/269)
	Chronic complications	84.4%(318/377)	63.9%(69/108)	92.6%(249/269)
	*Eye lesions*	84.9%(270/318)	60.9%(42/69)	91.6%(228/249)
	*Peripheral neuropathy*	77.0%(245/318)	79.7%(55/69)	76.3%(190/249)
	*Foot lesions*	20.8%(66/318)	4.4%(3/69)	25.3%(63/249)

#### Assessment of processes of care

All participants had received at least one blood glucose and blood pressure measurement within the previous year. However, no participant had received any blood cholesterol measurement. Less than 10% of participants had received at least one eye (9.0%) or foot (8.8%) examination in the previous year. Only 6.4% of participants (24/377) had been assessed for kidney damage using protein measurement in urine within the previous year. Details are shown in Table 5. With regard to diabetes education, over four fifths of clients had ever received T2DM education but, information about complications or danger signs was not commonly provided to clients (< 30%).

#### Assessment of health outcomes

Glycaemic control, was noted in 56.8% (214/377) of the clients; with blood glucose levels ranging between 3.4 and 32.2 mmol/L. Blood pressure control was found in 49.3% (186/377) of the clients. The proportion of clients that reported at least one complication was 84.4% (318/377). The most frequently reported complications were eye lesions and peripheral neuropathy 84.9% (270/318) and 77.0% (245/318) respectively. These outcomes were apparently better among clients seen at HC IVs compared to those seen at hospitals. Clients seen at HC IVs were more likely to have controlled blood glucose and pressure and were also less likely to report chronic complications compared to those seen at hospitals. Summary is shown in Table 5.

### Barriers to delivery of and access to diabetes care

Interviews with providers of diabetes care revealed a number of factors that affected service delivery and quality of care within the rural setting. Some of the challenges were uniform across all interviewed providers and others where more distinct to specific levels of care.

The most common cross-cutting barrier in the delivery of diabetes care was poor availability of diagnostic/monitoring equipment and essential medicines. All health workers interviewed in this study complained of recurrent stock outs of T2DM drugs and diagnostics especially test strips, which they felt contributed to poor management of patients and overall service delivery.

*When you don’t have the test strips for instance, you cannot tell the client’s blood glucose levels, so you end up just putting them on a regimen but when you are not sure if its adequate or not….. The issue of medicines also affects us. Sometimes a client comes and they need say metformin but we haven’t had metformin for a while so instead of sending the client away, you end up putting them on injectables instead of sending them away with nothing. (* **Clinical officer** *)*

Availability of medicines was also a serious challenge for the health workers. While some complained about metformin for T2DM, others were more concerned about insulin, which is mostly used in type 1 diabetes. Due to frequent stock-outs of metformin, health workers switched clients to insulin regimens. The frequent drug shortages in some facilities was attributed to high patient load.

*As we speak per now, there’s no even a single tablet of metformin. Instead, we have got injectables, which not all patients like but we have to push them on injectables. The reason, this hospital serves about 5 districts. I think the drugs would be enough but given the extended family, they end up not being enough. Service would be okay but the problem, the numbers are overwhelming.* (**Nursing Officer**)

Other barriers to quality that were reported by the health workers were: lack of training in standard T2DM care, inadequate number of clinicians and nurses in outpatient clinics at some facilities, high workload, lack of information/education/communication materials and lack of job aids for provision of standard T2DM care.

*We have an issue of the health workers. Most of our health workers here are not confident in diabetes care. I think may be the training is not adequate so you find that most tend to shy away from working on diabetes clients. (* **Medical Officer** *)*

Furthermore, while health workers reported having the basic Uganda clinical guidelines that some commended for guiding on diagnosis and treatment, others indicated that the guidelines were shallow. When asked about the adequacy of the treatment guidelines with respect to diabetes, one health worker explains that,

*I don’t think so, because it really doesn’t bring out this thing (treatment) clearly. Glibenclamide itself has been discouraged because if somebody has type2 diabetes and you just begin with glibenclamide, it has side effects like making somebody become obese, which would predispose this person to conditions like hypertension. So, the guidelines are not clear. Even the drugs are supposed to be given in a stepwise manner beginning with lower doses and increase gradually where necessary. Beginning with higher doses may contribute to resistance since the person is taking these drugs for a long time. (***Clinical officer**)

**Fig. 1:**
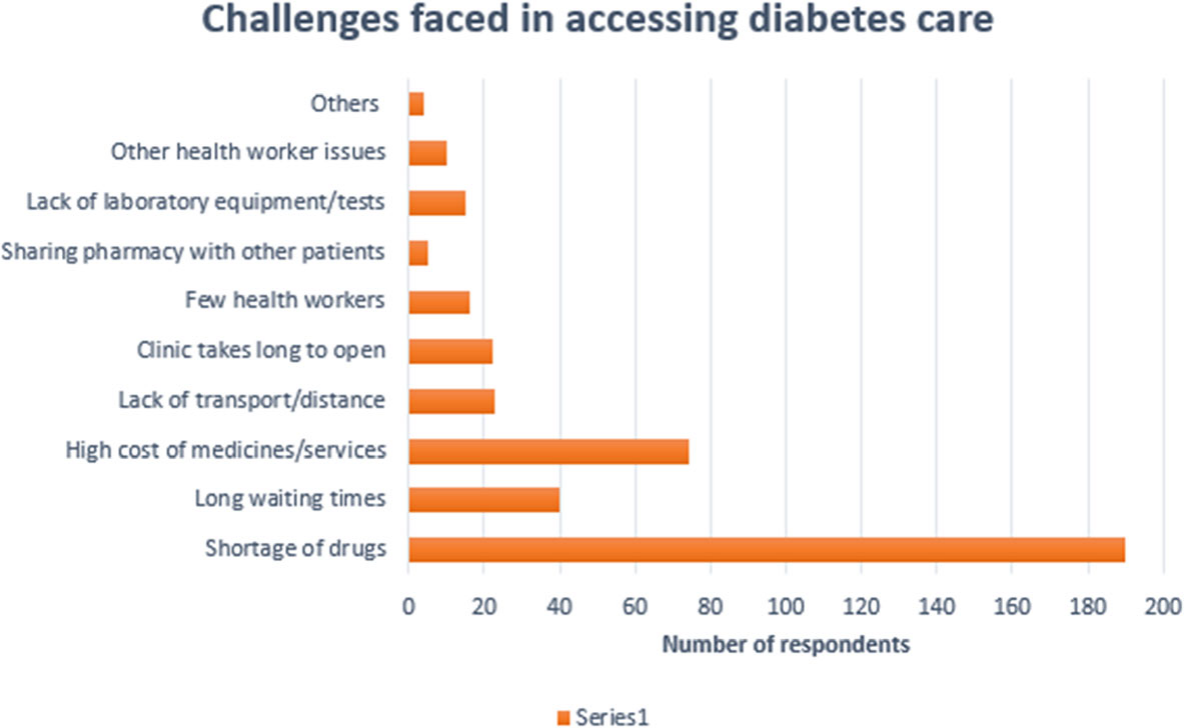
Challenges faced in accessing Diabetes care

On the other hand, from the clients’ perspective, the most frequently reported challenge faced in accessing care was the lack of drugs at the facilities (almost half of those interviewed 190/50.4%) Fig. 1. This forced clients to buy the necessary medicines from private outlets which was unaffordable to many. Other frequently reported challenges included: high cost of services such as paying for the glucose check and buying medicines due to stock outs in public facilities, lack of transport due to long distances to the facilities, long waiting times, sharing of service delivery points with other patients, late opening of the clinic, lack of laboratory equipment/supplies and inadequate number of health workers providing diabetes services at the facilities. Some clients also reported poor attitude and tardiness of health workers in reporting to work, as well as lack of specialists as barriers to quality care.

## Discussion

In this facility-based cross-sectional study, the quality of outpatient diabetes care in two rural districts in Uganda was assessed, using structural, process and outcome measures. This is one of the few studies that has conducted such an assessment in a context where systems for NCD care have only started gaining attention. The overall capacity of health facilities to deliver T2DM healthcare was rated above average, while process and outcome performance was poor. Health facility-related factors were the major barriers to the provision of and access to T2DM care.

The readiness of the health facilities in this study to offer DM services was less than optimal due to shortage of trained health workers, poor availability of supplies and medicines, inadequate guidelines and support systems for diabetes services. This finding is similar to what was reported by other, mostly cross-sectional studies, that assessed readiness and quality of diabetes care in Ethiopia [17], Bangladesh [18], Brazil [28] and Malawi [19]; where provision of diabetes services was greatly affected by lack of effective guidelines for diagnosis and management of diabetes, shortage of trained health workers and inadequate availability of medicines and equipment. In addition to these, a facility capacity assessment survey in Uganda [16] also found gaps in the availability of essential services and inadequate diagnostic capacity in most of the public sector facilities assessed. These contribute to low functional capacity to adequately deliver quality care, unmet health needs among clients, low motivation among health workers and delays in receiving quality care.

Nonetheless, the existing functional capacity (73.9%) observed in this study is an opportunity that can be leveraged to improve service availability. Our findings show that the facilities especially the hospitals in these rural settings have sufficient tools and resources in terms of equipment and diagnostic tests to provide essential diabetes services, despite irregularities in availability. The lower facilities also have some capacity but this needs to be improved especially with capacity of the healthcare providers and availability of medicines and diagnostic equipment.

The above gaps in foundation elements were also reported as major barriers to T2DM service delivery & use in this study. A qualitative study in the Netherlands among healthcare providers adds lack of patient motivation and lack of awareness of lifestyle programs and prevention initiatives among healthcare providers as barriers to service delivery [29].

The low screening rates for chronic complications of less than 10% found in this study may be attributed to gaps in service readiness and may result in late detection & management of preventable complications. This is likely to exacerbate T2DM-associated morbidity and high healthcare costs among affected individuals. Also, while random blood sugar is not the preferred test for blood glucose monitoring, it was the most frequently used test in facilities. Inability to conduct the recommended tests points to a gap in the of quality T2DM care. Similar patterns showing low rates of performing particularly kidney, eye and foot examinations have been reported elsewhere [19, 28, 30, 31].

Given the structural shortfalls and inadequate processes of care noted above, the outcomes of T2DM care were in this study found to be poor. Suboptimal outcomes have been reported by other studies, with the proportion of clients with controlled glucose ranging between 26.9 and 42.8% [20, 31–34]. Poorly controlled blood glucose and BP increase the risk for both macro- and micro- vascular complications. This may explain why over 80% of the participants in this study reported to have at least one T2DM-related complication. The prevalence of complications from this study (84.4%) is higher than 52.0 and 59.7% reported in in China [35] and Ethiopia [36] respectively; which were also cross-sectional hospital-based surveys, but used medical records review to identify presence of complications. Nonetheless, the studies do affirm that diabetic neuropathy and retinopathy are common complications among patients with T2DM, as found in this study. We recommend further investigation using more robust techniques to ascertain actual burden of these complications.

Taking both client and provider perspectives, this study provides important information on three dimensions of T2DM care in a rural setting which can be used to develop, implement and monitor quality improvement interventions. However, the study was limited by the use of self-reports which may have resulted in over- or under- estimation of some indicators. This was minimized by review of client records to verify some reports. The assessment of BP control based on a single set of BP readings may have resulted in under or over estimation of burden of high BP.

## Conclusions

The quality of T2DM care in this rural setting is inadequate. The poor process of care coupled with inadequacies in availability of medicines and basic technologies, training of health workers in standard diabetes care, lack of standard guidelines and weak support system for diabetes services are key hindrances to the attainment of desirable outcome targets and access to quality care. We recommend overall improvement in the process of T2DM care at all health facility levels including training of health workers in standard T2DM care, provision of standards for diagnosis and management of diabetes, regular mentorship and support supervision. Also, health facilities should be regularly and adequately stocked with essential medicines and key diagnostic tools for T2DM management; with well-defined information systems to track burden of T2DM and services delivered. Operational and implementation research to identify and assess impact of quality improvement interventions most feasible for this setting should be done.

## Supplementary information

**Additional file 1.** Data collection forms.

## Data Availability

The dataset supporting the conclusions of this article is available from the corresponding author on reasonable request.

## Abbreviations

BP: Blood pressure
HC: Health centre
IDF: International diabetes federation
IQR: Inter-quartile range
NCDs: Non-communicable diseases
OR: Odds ratio
RBS: Random blood sugar
SARA: Service availability and readiness assessment
SD: Standard deviation
T2DM: Type 2 diabetes mellitus
