# Supplementary material for: Quality and barriers of outpatient diabetes care in rural health facilities in Uganda – a mixed methods study
Source: BMC Health Serv Res. 2019 Oct 16;19:706. doi: 10.1186/s12913-019-4535-x (PMC6796349; doi:10.1186/s12913-019-4535-x)
Supplement: Supplementary file 1 — Additional file 1. Data collection forms. [file 12913_2019_4535_MOESM1_ESM.docx]

**Data collection forms**

**Form 1: Health facility quality assessment form**

For the study “Assessment of the Quality of Diabetes Care in healthcare facilities in a rural setting in Uganda”

Study number:_____________________________

Interviewer initials:_____________ Date of assessment (d/m/y):____/____/_______

**PART A: Facility identification**

| Name of facility |  |
| --- | --- |
| Level of facility |  |
| District |  |
| Contact person where possible (name/phone number) |  |

**PART B: Administrative indicators**

**B.1. Human resource for Diabetes care**

HR1. Discuss with the head of facility to determine the number of health workers who usually have diabetes care responsibilities. *Indicate not applicable (NA) if cadre is not suitable for level of facility*

| **Category** | **B1a). No. of HWs assigned to the facility** | **B1b). No. of HWs assigned to the facility who provide diabetes services** | **B1c). No. of HWs who provide diabetes services present on day of data collection*** | **B1d). No. of HWs who provide diabetes services trained in standard diabetes care** | **B1e). No. of HWs trained in standard diabetes care present on day of data collection** |
| --- | --- | --- | --- | --- | --- |
| Medical officers |  |  |  |  |  |
| Clinical officers |  |  |  |  |  |
| Nurses/MW |  |  |  |  |  |
| Pharmacists |  |  |  |  |  |
| Others (specify) |  |  |  |  |  |
| **Total** |  |  |  |  |  |

**B.2. Diabetes services**

DS1: How many days per week are diabetes services provided? Days/week:____________

DS2: Which diabetes services are provided at this facility? *Tick all applicable*

*Diabetes diagnosis(1), (2)monitoring (glucose/blood pressure/cholesterol), treatment(3), diabetes education(4), psychosocial support(5), screening for complications(6), laboratory services(7), (8)others (specify)__________________*

DS3: Which methods are used at this facility to diagnose diabetes?___________________

DS4: Are walk-in diabetes services provided at this facility? Yes(1) No(0)

DS5. How many times during the last six months did the facility receive a supervisory visit related to diabetes care from higher level? Times:____________

DS6. During the visit, what area(s) of diabetes care were assessed? _____________________

DS7. Where do you refer the severely-ill diabetes patients? *Tick appropriately*

(1) Hospital, specify name:_____________

(2) Private physician/clinic

(3) Other, specify:_____________________

DS8. What are the common reasons for referral of diabetes clients? __________________

DS9. Does the facility have a specific structured data capture system for diabetes services/care? Yes(1) No(0)

*If yes*, specify the system used (manual/computerized; structure/unstructured):_____________

Specify data captured:_________________________________

**Part C: Amenities**

A1. Is there a separate diabetes clinic in the health facility? ____Yes(1) ____No(0)

A2. Inspect the room (clinic space) for the following

|  | **Available** | |
| --- | --- | --- |
| A2a. Consultation table with stationary (investigation forms, referral forms, follow up forms, etc) | Yes(1) | No(0) |
| A2b. Nurse’s table for education, blood testing, etc |  |  |
| A2c. Nursing station for files & appointments |  |  |
| A2d. Diabetes education materials |  |  |
| A2e. Room with privacy for examination/counselling |  |  |
| A2f. Others (specify) |  | |

A3. Does the facility have accessible means of transport for patients requiring referral? Yes(1) No(0) if yes, specify transport means: ________________________________

A4: Does the facility have electricity? Yes(1) No(0) if yes, specify main source:________

A5: Does the facility have an improved water source (piped water/taps/borehole)? Yes(1) No(0) if yes, specify commonly used source: ___________________

A6: Does the facility have functional sanitation facilities available for outpatients? Yes(1) No(0) if yes, how many________________, specify type:________________________

A7: Does the facility have communication equipment (readily accessible phones/radio calls)? Yes(1) No(0) if yes, specify: _____________________________

A8: Does the facility have functioning computers and access to internet? Yes(1) No(0)

**PART D: Logistics and supplies**

**D.1. Equipment & Supplies**

*Ask a health worker to show you around the facility. Look and touch to complete the following questions.*

E1. Does the facility have the following equipment and supplies?

|  | **Available** | |  | **Functional** | |
| --- | --- | --- | --- | --- | --- |
|  | **Yes(1)** | **No(0)** | **NA** | **Yes(1)** | **No(0)** |
| 1. Accessible and working adult scale |  |  |  |  |  |
| 1. Blood pressure machine |  |  |  |  |  |
| 1. Stethoscope |  |  |  |  |  |
| 1. Blood glucose machine(s)-glucometer |  |  |  |  |  |
| 1. Glucose testing strips |  |  |  |  |  |
| 1. Measuring tapes |  |  |  |  |  |
| 1. Urine testing strips |  |  |  |  |  |

E2. Does the facility have guidelines and/or standards for diabetes services? Yes(1) No(0)

*If yes, which guidelines and/or standards are available*? _________________________

**D.2. Essential Medicines**

|  | Yes(1) | No(0) |  |
| --- | --- | --- | --- |
| **EM1.** Is there a pharmacy/dispensary in the facility? |  |  |  |
| **EM2.** Have the following diabetes drugs been stocked in the pharmacy in the past 12months? |  |  |  |
| EM2a. Insulin |  |  |  |
| EM2b. Metformin (500mg) |  |  |  |
| EM2c. Glibenclamide (5mg) |  |  |  |
| EM2d*. Others (specify)* |  |  |  |
| **EM6.** Stock situation of each of the above drugs in the past one month (***Verify with stock cards***) | In stock | Out of stock | Drug available today |
| EM6a. Insulin soluble |  |  |  |
| EM6b. Metformin (500mg) |  |  |  |
| EM6c. Glibenclamide (5mg) |  |  |  |
| EM6d. Others (*specify*) |  |  |  |

**D.3. Diagnostic capacity**

| DC1. Is there a medical laboratory in the facility? | Yes(1) | No(0) |  |
| --- | --- | --- | --- |
| DC2. Are any of the following laboratory tests done in the facility? | | |  |
|  | Yes(1) | No(0) | Not applicable(2) |
| *DC2a. Blood glucose (FBG/OGTT/HbA1c)* |  |  |  |
| *DC2d. Cholesterol* |  |  |  |
| *DC2e. Urine protein* |  |  |  |
| *DC2f. Albuminuria* |  |  |  |

**Form 2: Client Questionnaire**

For the study “Assessment of the Quality of Diabetes Care in healthcare facilities in a rural setting in Uganda”

Study number:_________________________

Interviewer initials:_______________ Date of interview (d/m/y): ____/____/_________

**Patient Identification**

| Respondent ID |  |
| --- | --- |
| Facility where interview is conducted |  |
| Level of facility where interview is conducted |  |
| Place where patient lives |  |
| District |  |

**Section A: Socio-demographic characteristics**

***To begin, I would like to ask you some general questions about your self***

1. Sex of respondent (*tick appropriate option*): ____Male(0) ____Female(1)
2. How old are you? (*Record age in completed years*):__________________
3. What is the highest level of education you attended?

| No education(0) | Primary(1) | Secondary(2) | University(3) | Other(4)- *Specify* |
| --- | --- | --- | --- | --- |

1. What is your religion?

| Catholic(0) | Protestant(1) | Muslim(2) | Pentecostal(3) | Other(4)- *Specify* |
| --- | --- | --- | --- | --- |

1. What is your marital status?

| Never married(0) | Married(1) | Living together (not married) (2) | Widowed(3) | Divorced(4) | Separated (5) |
| --- | --- | --- | --- | --- | --- |

1. What is your main source of livelihood?

| Not working (0) | Salary earner(1) | Wage earner(2) | Peasant farmer(3) | Self-employed (4) | Student(5) | Other (6)  *(Specify)* |
| --- | --- | --- | --- | --- | --- | --- |

**Section B: Illness and treatment history**

***Now, I would like to ask you some questions about your condition and its treatment***

1. When were you first diagnosed with diabetes? (*Month & year or year alone*): _________
2. How long have you had diabetes? (*Time in years*):__________________
3. Apart from diabetes, which other medical conditions do you suffer from? *Tick all applicable*

| None (0) | Hypertension (1) | Tuberculosis (2) | HIV/AIDS (3) | Cancer (4) | Other CVDs(5) | Other conditions(6)-*Specify* |
| --- | --- | --- | --- | --- | --- | --- |

1. When were you first started on treatment for diabetes? (*Month & year or year alone*):_____
2. How long have you been on treatment for diabetes? (*Time in years*): _________
3. How many types of medicines for diabetes are you taking? Number:_________
4. Which treatment regimen are you on? (*Ask for the patient’s treatment card and record*)

| **Drug** | **Is the medicine prescribed (*Tick*)** | | **Prescription (***write patient’s prescription for drug given***)** |
| --- | --- | --- | --- |
|  | **Yes(1)** | **No(0)** |  |
| (1)Metformin (alone) |  |  |  |
| (2)Glibenclamide (alone) |  |  |  |
| (3)Insulin (alone) |  |  |  |
| (6)Metformin + Glibenclamide |  |  |  |
| (7)Metformin + Insulin |  |  |  |
| (8)Metformin + Glibenclamide + Insulin |  |  |  |
| (9)Other OHAs (*specify*) |  |  |  |
| (10)Other medicines patient is taking- (*List*) |  | | |

1. Assess for presence of complications. Ask (Do you have any of the following complications?) and verify with patient’s records. *Tick appropriately all that apply*

| **Complication** | **Yes(1)** | **No(0)** |
| --- | --- | --- |
| **None(0)** |  |  |
| ***Eye lesions(1)***: visual impairment or blindness in one or both eyes |  |  |
| ***Foot lesions(2)***: having ulcers on the feet; or amputation not do due to accidents or other infection |  |  |
| ***Renal disease(3)***: experience of changes in urine output, swelling of extremities |  |  |
| ***Peripheral neuropathy(4)***: experience of pain, tingling, numbness or loss of sensation to common stimuli (heat, sharp objects, etc) |  |  |

**Section C: Knowledge of diabetes control and care (*circle multiple responses*)**

1. Do you receive information about your condition/treatment when you come to this facility?

Never(0) Always(1) Sometimes(2) **If Never, go to Q.19**

1. Did you receive information about your condition/treatment today?

Yes(1) No(0) **If No, go to Q.19**

1. Who provides the information?
   1. Professional health worker (doctor, clinical officer, Nurse, pharmacist)
   2. Village health worker
   3. A fellow patient
   4. Others, *specify* _______________
2. What type of information is given to you by that person? *Tick all that apply*
   1. How to prevent my condition & its complications: Yes(1) No(0)
   2. Factors that make my condition worse like excess alcohol consumption: Yes(1) No(0)
   3. Complications that can arise: Yes(1) No(0)
   4. Importance of regular check up by a professional: Yes(1) No(0)
   5. Information about the medicines am taking: Yes(1) No(0)
   6. Signs of serious disease: Yes(1) No(0)
   7. Monitoring glucose levels: Yes(1) No(0)
   8. Others (*specify*): _______________________________________________
3. Do you know that diabetes can make those affected to get other health problems?

Yes(1) No(0) **if No, go to Q.21**

1. If yes, what are some of those health problems a person with diabetes can get? *Tick all that apply*
   1. Heart problems
   2. Blindness
   3. Foot problems (ulcers, amputations)
   4. Kidney problems
   5. Infections
   6. Erectile dysfunction in men
   7. Others (*specify*): __________________________________________
2. For a person with diabetes, what can they do to stay healthy and prevent complications? *Tick all that apply*
   1. Eat healthy
   2. Exercise regularly
   3. Adhere to treatment
   4. Comply with recommendations from the health worker
   5. Avoid or reduce cigarette smoking
   6. Avoid or reduce alcohol consumption
   7. Going for regular medical check-up by a professional person
   8. Others (*specify*): __________________________________________________
   9. Don’t know

**Section D: Access and Service utilization**

1. How often do you go for general check-up at a facility in a year?

No. of times:___________________

1. How often do you get your blood glucose levels checked in a period of 3 months?

No. of times:___________________

1. How long does it take you to reach this facility on foot? Time:____________
2. When you come to this facility, what services do you commonly get? *Tick all that apply*
   1. General check-up
   2. Blood pressure measurement
   3. Blood glucose measurement & other laboratory investigations
   4. Eye check up
   5. Foot examination
   6. Diabetes education
   7. Treatment/medication
   8. Others (*specify*): ____________________________________________
3. Whenever you come to the facility, do you get all the services that you require?

Never(0) Always(1) Sometimes(2)

1. Do you pay for any diabetes services at this facility? Yes(1) No(0) **if No, go to Q.28**

If yes, which services do you pay for? _________________________

Is the cost affordable to you? Yes(1) No(0)

1. Have you encountered difficulties accessing diabetes services when you need to?

Yes(1) No(0) **if No, go to Q.29**

If yes, what kind of difficulties? ______________________________________________

1. In your opinion, what factors affect the quality of diabetes care you receive at this facility?

_______________________________________________________________________

1. What can be done to improve quality of care for people with diabetes?

_________________________________________________________________________

**Section E: Investigations & measurements**

1. Indicate whether following tests were done and the findings. Ask the patient if they have had the tests done and ask for their medical records to fill the following process & outcome measures

**Processes of diabetes care**

| **Indicator** | **Has the test been done in the past 12 months?** | | **Number of times test was done in previous year** | **Dates & values of measurements done?**  **(value & date)** | |
| --- | --- | --- | --- | --- | --- |
|  | **Yes(1)** | **No(0)** |  | **Value** | **Date** |
| Blood glucose(1) |  |  |  |  |  |
| Blood pressure(2) |  |  |  |  |  |
| Cholesterol(3) |  |  |  |  |  |
| Protein(4) |  |  |  |  |  |
| Albumin(5) |  |  |  |  |  |
| Eye examination(6) |  |  |  |  |  |
| Foot examination(7) |  |  |  |  |  |

**Outcomes of diabetes care**

**Glucose control**

| GC1. Was measurement taken on current visit? | | GC2. If Yes, record value (indicate measure: FPG/HbA1c/RBS) | GC3. If No, has measurement been done within past 3 months? | | GC4. If yes, record values and dates when measurement was done in past 3 months | |
| --- | --- | --- | --- | --- | --- | --- |
| Yes(1) | No(0) |  | Yes(1) | No(0) | Value | Date |
|  |  |  |  |  |  |  |

**Blood pressure control**

| BP1. Measurement taken on current visit? Record value [SDP/DBP] | |
| --- | --- |
| SDP | DBP |
|  |  |

**Form 3: Key Informant Interview guide**

For the study “Assessment of the Quality of Diabetes Care in healthcare facilities in a rural setting in Uganda”

Interviewer initials: …………………... Date of interview (d/m/y): ……/…../……….

**Background information**

| Respondent ID |  |
| --- | --- |
| Sex (*tick as appropriate*) | Male Female |
| Age (*in completed years*) |  |
| Type of provider (*Cadre*) |  |
| Name of facility where provider works |  |
| Level of facility where provider works |  |
| Highest level of education attained |  |
| Major aspect of diabetes care involved in |  |

1. For how long have you provided diabetes care at this facility? Time …………………
2. Have you ever received training in quality diabetes care, beyond the training in school?

*If yes, when was the training attended? Who provided the training? Which type of training was it?*

**Structural, process and perception of quality of diabetes care**

1. What services do you commonly provide to people with diabetes? *What factors influence the services you provide to a person with diabetes? Do you think that these services are adequate? Why? What key services should be available for all diabetes patients?*
2. What is your opinion on the capacity and readiness of this facility to provide standard diabetes care? *Which key aspects are inadequate?*
3. What is your opinion regarding the adequacy of the current Ugandan guidelines for provision of diabetes services? *Which areas need improvement*?
4. What is your opinion on the quality of diabetes care provided at this facility? *What factors do you think affect the quality of diabetes care provided at this facility? What is your opinion on the diabetes care packages currently provided?*
5. What challenges have you encountered during provision of diabetes services? *How can these be addressed?*
6. What can be done to improve the quality of care provided to people with diabetes?

This is the end of the interview. Thank you for your time
